# Optical and Electrical Properties of TTF-MPcs (M = Cu, Zn) Interfaces for Optoelectronic Applications

**DOI:** 10.3390/molecules201219742

**Published:** 2015-11-25

**Authors:** María Elena Sánchez-Vergara, Mariel Leyva-Esqueda, José Ramón Alvárez-Bada, Verónica García-Montalvo, Iván Darío Rojas-Montoya, Omar Jiménez-Sandoval

**Affiliations:** 1 Facultad de Ingeniería, Universidad Anáhuac México Norte, Avenida Universidad Anáhuac 46, Col. Lomas Anáhuac, Huixquilucan 52786, Estado de México, Mexico; mariel.leyva1901@gmail.com (M.L.-E.); ramon.alvarez@anahuac.mx (J.R.A.-B.); 2 Instituto de Química, Universidad Nacional Autónoma de México, Circuito Exterior, Ciudad Universitaria, México 04510, D.F., Mexico; vgm@unam.mx (V.G.-M.); ivandachem@hotmail.com (I.D.R.-M.); 3 Centro de Investigación y de Estudios Avanzados del Instituto Politécnico Nacional, Unidad Querétaro, Apartado Postal 1-798, Querétaro, Qro. 76001, Mexico; ojimenez@cinvestav.mx

**Keywords:** phthalocyanines, thin films, organic semiconductor, optical properties, electrical properties

## Abstract

Sandwich structures were fabricated by a vacuum deposition method using MPc (M = Cu, Zn), with a Tetrathiafulvalene (TTF) derivative, and Indium Tin Oxide (ITO) and aluminum electrodes. The structure and morphology of the deposited films were studied by IR spectroscopy, scanning electron microscopy (SEM) and energy dispersive spectroscopy (EDS). The absorption spectra of TTF derivative-MPc (M = Cu, Zn) thin films deposited at room temperature were recorded in the spectral range 200–1000 nm. The optical band gap of the thin films was determined from the (α*h*ν)^1/2^ *vs.* *hν* plot. The direct-current (DC) electrical properties of the *glass/ITO/TTF_deriv_-MPc (M = Cu, Zn)/Al* structures were also investigated. Changes in conductivity of the derivative-TTF-enriched Pc compounds suggest the formation of alternative paths for carrier conduction. At low voltages, forward current density obeys an ohmic *I*-*V* relationship; at higher voltages, conduction is mostly due to a space-charge-limited conduction (SCLC) mechanism.

## 1. Introduction

The discovery of organic light-emitting diodes (OLEDs) based on organic molecules has led to a fair amount of work devoted to understand the optical transport properties of organic semiconductors (OS). The color of the emitted light depends on the precise composition of the OS. Red-, green-, and blue-emissive OS can be combined to produce the full-color spectrum [1]. As OS thin films are composed of individual molecules attracted by weak van der Waals forces, many of the physical properties of the solids have a strong dependence on the extent of intermolecular orbital overlap [2]. Organic thin films’ properties can benefit from achieving vertical molecular alignment with molecules standing up, or edge-on, so that the π-π stacking direction is parallel to the substrate as well as the conducting channel [2]. Furthermore, optical properties, such as absorption and emission, are most often anisotropic, a characteristic which has been exploited for OLEDs as well as for organic solar cells (OSC) [2]. An OSC is constituted by an OS that acts like an active layer between an anode and a cathode. OSCs are heterostructured by the anode, which usually is a transparent conductor oxide deposited on a substrate (usually glass, although it could be another kind of material that acts as protection and support of the device and is transparent, in order to allow the entry of sunlight to the active layer). The basic processes occurring in the organic solar cells can be described in terms of five steps: excitation, exciton formation, exciton diffusion, exciton dissociation, and charge collection. Upon attachment of an OS sample between the two electrodes, an energy barrier is formed. The energy barrier stimulates exciton dissociation and separates the charges that compose them. Delays in exciton dissociation due to geometrical or mobility factors may lead to charge recombination, which decreases cell efficiency. In order to reduce recombination and further increase light absorption, structures with two or more thin layers of OS have been created. A plurality of layers may distribute the energetic stepping to enhance excitonic dissociation and charge extraction. Layers can be formed of *n* (acceptor) or *p* (donor) materials.

Phthalocyanines (Pcs) and metallophthalocyanines (MPcs) are a class of highly stable OS, which are (with some exceptions) classified as *p*-type semiconductors with low mobility and carrier concentration, with absorption bands extending from the ultraviolet to the infrared region [3,4]. It has been established that, when suitable electrode materials are employed, the formation of a Schottky-type contact between a metal electrode and a Pc-MPc is possible [4]. The electrical properties of Pc-based devices are also known to depend on the electrode material employed and ambient conditions [4]; they exhibit changes in conductivity when exposed to oxygen and hazardous gases, such as NO_2_ and NH_3_ [5]. In MPc thin films, conductivity is ohmic at low applied voltages, whilst, at higher voltages, it is better described in terms of a space-charge-limited current (SCLC). The latter process has been described in terms of an exponential trap distribution [5]. The steady-state transport properties of carriers through OS are dominated by the presence and energy distribution of carrier-trapping sites [6]. In the case of CuPc, the most widely studied material of this class, it is well known that traps exist in both single crystals and thin films at a single energy level [6]. Transport properties of MPcs, are strongly related to the valence band electronic configuration and the excitation-deexcitation process between highest occupied molecular orbitals (HOMO) and lowest unoccupied molecular orbitals (LUMO) [7]. For example, the F_16_CuPc complex is used as an electron acceptor in solar photovoltaic cells; the complex energy locations are HOMO at −6.3 eV and LUMO at −4.9 eV [8]; similarly, the calculated HOMO-LUMO gap for NiPc has been found to be 2.41 eV [9]. In general, for organic electronic materials, the behavior of a single electron, including electron transfer/transport, *n-*/*p-* doping processes, and so forth, depends primarily on the energy of the HOMO and LUMO levels and the orbital interactions [10]. The molecules with a controlled HOMO/LUMO gap are the prime targets for electronic applications. Tetrathiafulvalene (TTF) and its derivatives are capable of generating structures with small HOMO/LUMO gaps [10]. MPcs have long been known to undergo electron-transfer reactions, both to and from their excited states, and strong electron donors such as TTF [11]. Pc is an acceptor moiety for TTF−σ-acceptor conjugates that was first used by Bryce *et al.* [10]. TTF, an organic donor, represents the prototypical molecular unit used in molecular conduction. The good donor properties of TTF are partly due to aromatization energy gain when going from the dithiolylidene moiety in the neutral molecule to dithiolium aromatic rings in the oxidized states [10]. The chemical functionalization of TTF provides the opportunity to correlate the collective electronic properties of their charge transfer salts with precise structural features in order to ultimately be able to control and manipulate both the structure and the properties of the system [12]. Compounds containing eight TTF units on the periphery of the Pc macrocycle have resulted in amphoteric redox behavior: two oxidation waves of TTF and two reduction waves of Pc were observed and a gap of 1.25 eV could be deduced from the redox potentials [10]. Some TTF-MPcs systems have been synthesized [11,12,13,14,15,16], though only the oxidation and gap features of TTF-Pc have been studied [10].

The knowledge of the optical gap and electrical behavior of TTF-MPc thin films is important in many applications, such as solar energy, photo-conductivity, OLED, and numerous other applications. The aim of this work is to investigate the electronic structure, the transfer mechanisms and the optical gap of MPc (M = Cu, Zn) thin-film structures fabricated by vacuum thermal evaporation. We have also investigated the effect of doping these MPc compounds with a TTF-derivative (4,4′,5,5′,6,6′,7,7′ octahydro-dibenzo-tetrathiafulvalene). The MPcs (M = Cu, Zn) were selected among the Pc compounds because of their high chemical and thermal stability, high charge mobility and an effective nonlinear optical response [17,18]. We chose the TTF derivative because of its well-known ability to produce molecular assemblies with a small gap [14,15]. Structural and morphological properties of the thin films were examined by scanning electron microscopy (SEM), energy-dispersive X-ray spectroscopy (EDS) and FT-IR spectroscopy. This work also reports the optical parameters related to main transitions in the UV-Vis region, as well as the corresponding HOMO/LUMO gap calculations for TTF_deriv_-MPcs (M = Cu, Zn) thin films. As a sandwich-like configuration is frequently used in the preparation of thin-film diodes and transistors [3], the sandwich-type *glass/ITO/TTF_deriv_-MPc (M = Cu, Zn)/Al* structure was fabricated by vacuum thermal evaporation in two different evaporation ports, working consecutively with two crucibles of the same material and conformation. The TTF and MPc compounds had a 1:1 stoichiometric ratio. The first compound to evaporate and deposit over the glass/ITO substrate was TTF. MPc was then evaporated over the same substrate as TTF and with the same operating parameters. This allowed us to evaluate a diverse repertoire of electronic properties of MPcs arising from their large π-conjugated systems, as well as their outstanding propensity to assemble into cofacially-stacked arrays [15]. Transport characteristics of organic sandwich-like devices were studied from DC electrical measurements. The effect of thermal annealing on the electrical properties of thin sandwich systems was also investigated.

## 2. Results and Discussion

### 2.1. Structural Characterization

The comparison between the IR spectra of the synthesized powders and those of the deposited films indicate that there were no significant chemical changes in these materials during the thermal evaporation process to obtain the films. Thus, the deposited films are formed by the same macro-ions as those of the original synthesized powders. Table 1 shows selected signals corresponding to the synthesized powders and the thin film deposited onto a silicon substrate [19,20,21,22]: it was intended that the band assigned to the stretching vibration for the pyrrole be around 1065 cm^−1^, the bands responsible of the isoindole plane and carbon-nitrogen stretching occur around 1288 and 761 cm^−1^, respectively, the band around 1164 cm^−1^ be the result of the isoindole deformation and C-H out of plane bending of aromatic compounds at 620 cm^−1^ [20,21]. Additionally, the IR absorption technique was used to identify the phase nature of the MPc powder and thin film, as the IR spectrum is markedly dependent on chemical composition and crystalline form [21]. MPcs are known to have different polyforms, which can be identified by the IR absorption technique [23,24]. Two polymorphs (α-metastable and β-stable) can occur because of the slight differences in the π-π electronic interactions between the neighboring molecules in the lattice [21]. The spectral characteristics distinguishing the different MPc crystalline forms are located between 800 and 700 cm^−1^. The β-form gives rise to vibrations at about 770 cm^−1^, while the α-form produces vibrations at about 720 cm^−1^ [23,25]. From these studies, we are able to determine the phase and any significant chemical changes which may occur in these materials during the thermal evaporation and after annealing at 353 and 393 K for one hour. Table 1 shows the infrared absorption spectra of powder, as-deposited films and annealed films. Initially, both spectra of pellets CuPc and ZnPc pellets showed the two crystalline forms. When the spectra of the deposited MPcs were analyzed, α and β forms were observed with the values of 720 and 779 cm^−1^, respectively, for CuPc, and only 775 cm^−1^, corresponding to the β-form, for ZnPc. Apparently, the ZnPc material-to-substrate thermal gradient is responsible for the phase β nucleation, owing to the intramolecular forces of the atoms being rearranged. After annealing ZnPc at 353 and 393 K, no trace of the α-form was observed, but the β-form remained at 775 cm^−1^ (see Table 1). After the CuPc film was annealed at 353 and 393 K, the spectra show that the crystalline structures α and β remain. Table 2 shows the thicknesses of the two films, the copper-based being considerably larger. Due to the relatively large thickness of the TTF_deriv_-CuPc film, heating is not enough to mobilize the molecules. Thus, these molecules cannot be rearranged in a new crystalline structure and these results show that the earlier CuPc structure remains after the heat treatment [26,27]. During annealing, the film was heated below its melting/decomposition temperature for one hour. Although temperature and annealing time were both sufficient to raise the temperature of the entire film and promote a change in its crystalline structure, the presence of TTF derivatives seems to inhibit the nucleation of new grains or kernels that could modify the MPc structure; only a modest rearrangement of the molecules comprising the films was found.

**Table 1:** Characteristic FT-IR signals for TTF-MPcs (M = Cu, Zn) in pellets and thin films (cm^−1^).

Sample	ν(C-C) Isoindole Deformation	ν(C-N) Stretch in Pyrrole Ring	ν(C-N) in Plane Isoindole and Stretching Vibration	ν(C-H) in Plane Bending	Benzene Ring	TTF
CuPc-TTF Pellet	1611	1065	1288, 761	1421	697	1508, 715, 694
CuPc-TTF Thin Film	1596	1079	1287, 751	1437	697	1508, 721
CuPc-TTF (annealing 353 K)	1605	1080	1283, 774	1438		1508, 726
CuPc-TTF (annealing 393 K)	1596	1079	1287, 751	1437	697	1507, 721
ZnPc-TTF Pellet	1606	1075	1278, 750	1409	695	1500, 778, 683
ZnPc-TTF Thin Film	1596	1108	1284, 750	1420		1508, 778
ZnPc-TTF (annealing 353 K)	1609	1111	1284, 748	1422		1508, 779
ZnPc-TTF (annealing 393 K)	1609	1110	1284, 749	1424		1508, 774

**Figure 1:**
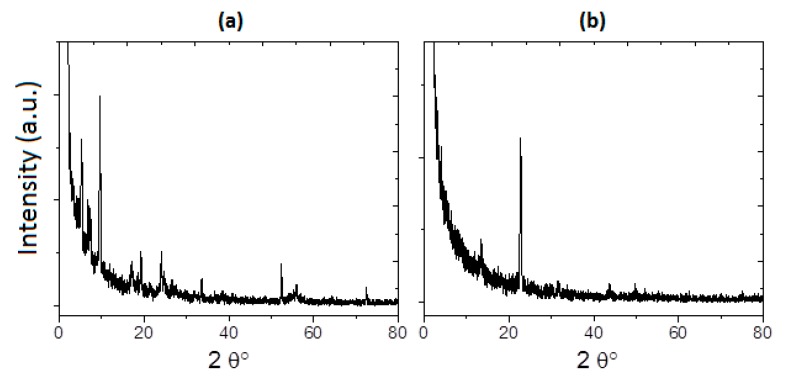
XRD for (**a**) TTF_deriv_-CuPc and (**b**) TTF_deriv_-ZnPc thin films at 393 K.

Molecular semiconductors are frequently produced as crystals; thus, X-ray diffraction (XRD) was undertaken to structurally characterize the deposited materials. The XRD patterns obtained for TTF_deriv_-CuPc in the thin-film form after annealing (393 K) are shown in Figure 1a; they indicate a polycrystalline structure and a mixture of α- and β-forms. Figure 1b shows the diffraction peaks of the corresponding XRD pattern from the TTF_deriv_-ZnPc film; only two significant peaks around 2θ° = 13.5° and 22.74° are found for the β-form. Accordingly, the IR analysis of the annealed thin films showed that the α- and β-crystalline structures of CuPc remain. Regarding ZnPc, no α-form was found after annealing, but the β-form persisted [2,21,28].

### 2.2. Microscopy Study

**Figure 2:**
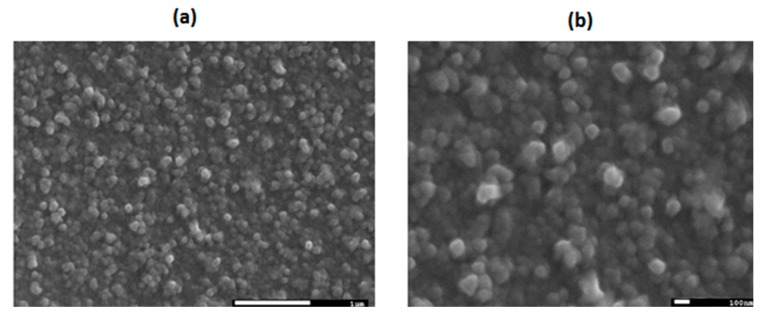
SEM images of TTF_deriv_-CuPc thin films deposited onto silicon wafers, at amplification (**a**) 25,000× and (**b**) 50,000×.

In both films, the main change in the crystal structure occurred during deposition, which provided a large amount of energy to the compounds. Most of this energy was converted into heat, which allowed film deposition. This should also have facilitated recrystallization during the annealing process. Nevertheless, the addition of TTF molecules hindered molecular motion and MPcs recrystallization. Film heterogeneity also affected the annealing process and transport properties. SEM was thus performed in order to ensure homogenous films. The SEM micrographs in Figure 2 show the surface morphology of TTF_deriv_-CuPc thin films (deposited on silicon) at 25,000× and 50,000× magnifications, respectively. From these micrographs, a homogeneous film morphology is observed. All images have granular features; the thin films have grain sizes within the range of 50 nm to 100 nm. The size and distribution of the grains also show a compact deposition. The homogenous and compact form of the films, consisting of relatively small grain particles, favors charge transport through TTF_deriv_-CuPc. On the other hand, the TTF_deriv_-ZnPc thin film showed little tendency to adhere to the substrate. This led to ZnPc producing an irregular layer when deposited upon TTF.

Roughness is another aspect to consider, and it must be low if we want to keep TTF_deriv_-MPc interaction and electronic transport. The roughness of the thin films onto quartz was examined by profilometry. RMS values are shown in Table 2. A larger roughness is observed for the TTF-CuPc film, which also happens to be the thickest one. Owing to the fact that the conditions for deposition were the same for both film samples, it follows that the difference in roughness values may be related to the different metallic atom in the Pc molecule, film thickness and the electrostatic forces occurring during deposition. High roughness values of the TTF_deriv_-CuPc film may lead to more scattering sites for the mobilized electrons, thus increasing electrical resistivity.

**Table 2:** Characteristic parameters of the TTF-MPc films.

Thin Film	Film Thickness (Å)	RMS Roughness (nm)	Indirect Fundamental Energy Gap, E_g1_ (eV)	E_g2_ and E_g3_ (eV)
TTF_deriv_-CuPc	11,667	1700	1.4	1.5, 2.8
TTF_deriv_-ZnPc	1620	66	1.7	1.3, 2.0

**Figure 3:**
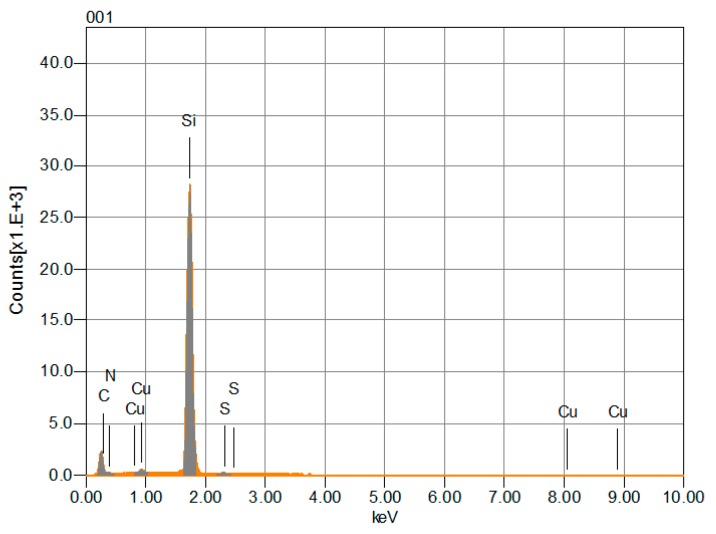
EDS spectra for the TTF_deriv_-CuPc thin film deposited on a silicon wafer.

The films were also analyzed by energy dispersive spectroscopy (EDS). The results for the TTF_deriv_-CuPc thin film are shown in Figure 3. This film has the expected chemical composition, as it consistently includes all of the expected elements for all the explored areas. The presence of N, Cu in TTF_deriv_-CuPc is attributed to the MPc part of the material, whereas the presence of S is attributed to the TTF derivative. These results confirm the fact that no chemical change of the material occurred during evaporation and can be complemented by those obtained from IR spectroscopy. The deposited films are formed by the same compounds as those of the original synthesized powder, as determined by comparison between the positions of the absorption bands in the spectra of the synthesized powders and those of the deposited films.

### 2.3. Optical Properties

**Figure 4:**
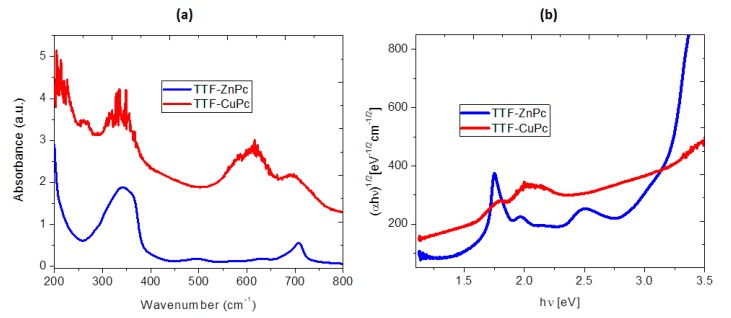
(**a**) Absorption spectra of the MPc and TTF_deriv_-MPc thin films and (**b**) plot of (α*h*ν)^1/2^ *vs.* photon energy *hν* of the TTF_deriv_-MPc films.

Optical absorption measurements are widely used to characterize the electronic properties of the materials through the determination of parameters describing the electronic transitions, such as the band gap [27]. The optical absorbance spectra of the TTF_deriv_-CuPc and TTF_deriv_-ZnPc thin-films deposited on quartz were recorded from 200 to 1000 nm and are shown in Figure 4a. The changes in the UV-Vis spectra are due to the contributions of the central atom in the Pc ring to the molecular orbitals in this region and the arrangement of the TTF units. The absorption peak around 272 nm, attributed to the TTF derivative, is not found in TTF_deriv_-ZnPc, suggesting a stack-like arrangement of TTF units while, in the region between 250 and 500 nm, the spectrum of TTF_deriv_-CuPc shows superimposed bands of TTF and CuPc species [13,15].

The UV-Vis spectra for MPc arise from molecular orbital interactions within the 18-π electron system and orbital overlap at the central metallic atom. Pcs have two absorption bands, the *Q* band, located in the visible region (530–800 nm) and the *B*, also called *Soret*, band, in the near-UV region (300–400 nm) [3,23,27,28,29,30,31]. It can be seen that there is an absorption peak at 334 and 343 nm for TTF_deriv_-CuPc and TTF_deriv_-ZnPc, respectively, which represents the π→π* transitions corresponding to an intense *Soret* band [27]. The absorption spectra occurring in the high-energy region of the *Soret* band indicate the presence of a d-band related to the central metallic ion. There are also two peaks at wavelengths of 616 and 696 nm for the TTF_deriv_-CuPc and 630 and 707 nm for the TTF_deriv_-ZnPc thin films, corresponding to an intense *Q* band which gives the onset energy [27]. The high-energy peak of the *Q* band has been assigned to the first π→π* transitions on the phthalocyanine macrocycle and the low energy peak has been variously explained as a second π→π* transition, as an exciton peak, as a vibrational interval and as a surface state [23]. The spectral distribution of the absorbance and the absorption coefficient of the investigated films, characterized by distinct peaks in the visible region, has generally been interpreted in terms of π→π* excitations. According to Cook *et al.* [14], the *Q* band around 696–707 nm is indicative of an enhanced aggregation of the Pc moieties in the films. From the absorption spectra, we suggest that the interactions between TTF and MPc are predominantly of an intermolecular character and exhibit the aggregation of MPc. The intermolecular spacing between the Pc rings is comparable with the intermolecular spacing between TTF molecules in the highly conducting complex with TCNQ, which could promote favorable intermolecular orbital overlap and inter-stack interactions [14].

Most of the TTF derivative applications are determined by the donor capabilities of this molecule, which are the result of the high-lying HOMO. The LUMO energy in such compounds is also rather high, as good donors are usually poor acceptors. Some TTF derivatives show an HOMO-LUMO gap of between 1.1 and 2.32 eV [10]: MPc as an acceptor moiety for TTF-σ-A conjugates shows an HOMO-LUMO gap of between 1.2 and 2 eV. The band gap is an important parameter in the physics of semiconductors and was determined for these materials from the Tauc model. The optical gap associated with each thin film is evaluated from the extrapolation of the linear trend in the spectral dependence of (αhν)^n^ *vs.* *hν* [31,32,33]. The absorption coefficient α is determined from the Lambert-Beer law and the plot of the optical transmittance [34,35]. The optical absorption coefficient of a material characterizes the exponential decay rate of light intensity as it traverses the solid [25]. Absorption (α ≥ 10^3^·cm^−1^) is related to direct interband transitions where the lowest part of the valence band and the highest part of the conduction band share the same wave vector, with an allowed transition across the band gap [23]. The absorption coefficient α is exponentially related to photon energy, in accordance to the empirical relation [32,33,35]:

(1) $$\alpha \text{hv = }{\beta ({\text{hv − E}}_{\text{g}})}^{\text{n}}$$

Factor β depends on the transition probability and is considered constant within the range of optical frequencies. For allowed direct transitions, *n* = 1/2 and, for allowed indirect transitions, *n* = 2. In crystalline semiconductors, direct transitions are, to a first approximation, the dominant contribution to optical transitions. However, as can be seen in Figure 4b and for all the thin films, indirect transitions (*E_gi_*) are related to a more linear behavior for the referred curves. The indirect electronic transitions seem to be of the π to π* type. In the electronic transitions from valence-band states to conduction-band states, there is no electronic momentum conservation [36]. Figure 4b shows the dependence of the absorption coefficient on photon energy; the optical gap determination is shown in Table 2. The indirect Tauc gap values of TTF_deriv_-CuPc and TTF_deriv_-ZnPc were found to be 1.4 and 1.7 eV, respectively. In general terms, Figure 4b shows that the curves for all materials are characterized by three portions with different slopes; thus, three activation energies are obtained and given in Table 2. E_g1_ is the intrinsic energy gap and E_g2_ and E_g3_ are the activation energies needed to excite the carriers from the corresponding trap levels to the conduction band and are associated with impurity conduction [3]. The fact that more than one linear portion can be found in Figure 4b indicates the existence of trap levels in the energy band gap; the tails associated to both the onset edge and fundamental edge are attributed to the phonons-assisted indirect transitions. The lower Tauc gap for TTF_deriv_-CuPc is related to several factors, such as: (i) the electronic configuration of copper(II) in the phthalocyanine which, when d^9^ is compared to d^10^ for zinc, allows for a higher electron mobility; (ii) the size differences of the metal atoms influence not only the electronic mobility within the molecule, but also the overlap and stacking within the TTF derivative; and (iii) finally, the crystalline structure of the MPc is related to the charges’ mobility, as the combination of α and β forms in CuPc apparently permits an easier electronic transport than in the case of a unique β form. The main difference between these two phases is the angle formed between the symmetry axis and the stacking direction. Alpha crystals have angles of 26.5°, whereas beta crystals have angles of 45.8°. The combination of these two crystalline forms may facilitate the ordered arrangement of CuPc with the TTF molecules.

### 2.4. Electrical Properties

**Figure 5:**
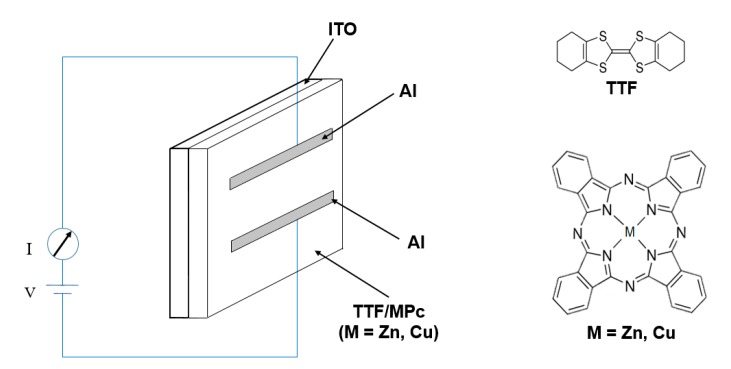
Chemical structures of MPc and TTF derivative and Schematic structure of device.

In order to evaluate the properties of the thin films for their use in organic devices, a series of four-layer, two-terminal devices were fabricated, each with a *glass/ITO/TTF_deriv_-MPc (M = Cu, Zn)/Al* structure (see Figure 5).

**Figure 6:**
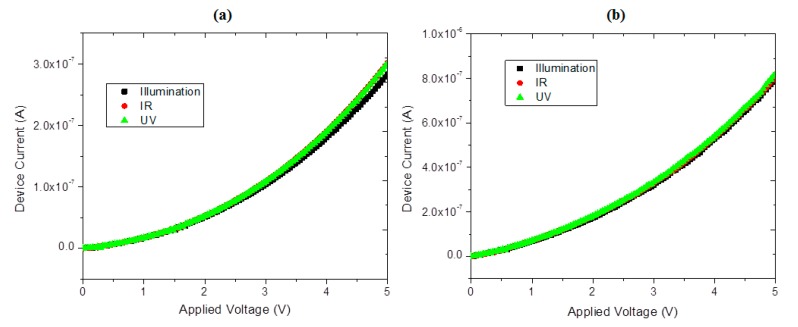
Current-voltage characteristics of the *glass/ITO/TTF_deriv_-CuPc/Al* device (in air) under white, infrared and UV illumination: (**a**) ITO is positively biased and (**b**) ITO is negatively biased.

Figure 6 shows the *I-V* characteristics for the sandwiched Cu device under three illumination types (white, infrared and UV light). No difference between IR and visible light is found in the *I-V* characteristic, as expected, with a mild increase in electrical current in the UV case due to the excitation of a relatively small number of charge carriers. Regardless of the wavelength of the incident radiation, the device follows the same overall behavior. The *I-V* characteristics display an asymmetric behavior in the ITO/*TTF_deriv_-CuPc/Al* case, with a larger device current occurring for negatively-biased ITO. The almost 3-fold increase in current may be related to the *n*-type semiconducting behavior of ITO and the donor character of TTF, which makes it behave as a *p*-type semiconductor. Forward and reverse biases thus arise from changes in ITO applied voltage. The fact that an increasing current with increasing applied voltage is to be found in both directions suggests that other processes (*i.e*., hole injection from ITO or from Al electrodes, as well as contributions from the MPc) are also occurring within the device. Similar results were obtained for the zinc device. The indirect transitions taking place in the films, apparently of ordered structure, as well as the weak response to UV radiation, may be due to the presence of TTF molecules disturbing the periodicity of the net and the formation of localized states that contribute weakly to conductivity. Moreover, as the process is controlled by electron hopping, the energy of the electromagnetic radiation (photons) is insufficient to excite large numbers of electrons to extended states above the conduction band. Thus, the contribution of external UV radiation to conductivity, while not negligible, is small compared to other factors.

**Figure 7:**
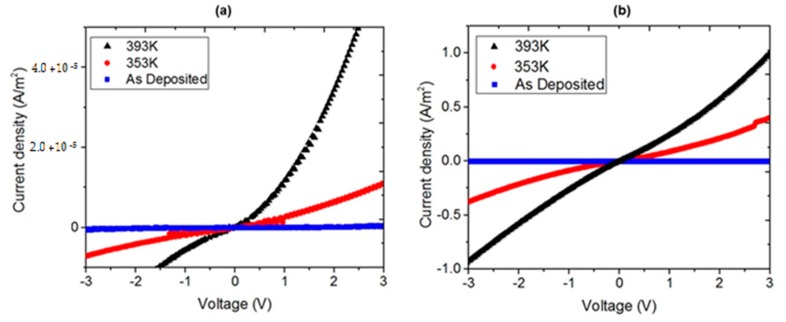
*J*-*V* characteristics of (**a**) CuPc and (**b**) ZnPc devices at different annealing temperatures.

The injection, charge and transport processes of MPc-based devices can be affected by annealing treatment [27]. In order to investigate the electrical properties of the devices’ junction, the dark *J*-*V* characteristics for various annealing temperatures have been evaluated and shown in Figure 7. The annealing affects the *J*-*V* characteristics of the MPc devices, as the current density was greater for annealed samples than for the as-deposited samples. According to Figure 7, the annealing temperature also affects the current *J*-*V* characteristics of the devices. An increase in the annealing temperature permits a molecular reordering of the TTF and MPc molecules that is related to thin-film uniformity and helps to reduce trap density in the structure, increase charge carrier mobility, and thus facilitate the injection and transportation of charge carriers. Indeed, an increase in the current density of the annealed samples with respect to the originally deposited ones could be found; practically no charge transport was found in the CuPc and the ZnPc devices without annealing. The MPcs *J*-*V* characteristics at room temperature can be described in terms of mild ohmic conduction in the lower voltage range, followed by SCLC (space-charge-limited conductivity), which is governed by an exponential trap distribution, in the higher voltage range [5,6,37]. The electrical results thus obtained suggest the existence of ohmic interfaces with a limited amount of charge transfer and band-bending, as would be expected of a junction made of a *p*-type semiconductor in contact with conducting interfaces (unlike Schottky barriers, typically involving *n*-type semiconductors). The addition of the TTF-derivative compound to the MPcs leads to a remarkable increase in conductivity, which can be attributed to the generation of conducting states in the corresponding band locations of both materials, which in turn may be explained in terms of orbital overlaps facilitated by the physical proximity of both molecular types.

**Figure 8:**
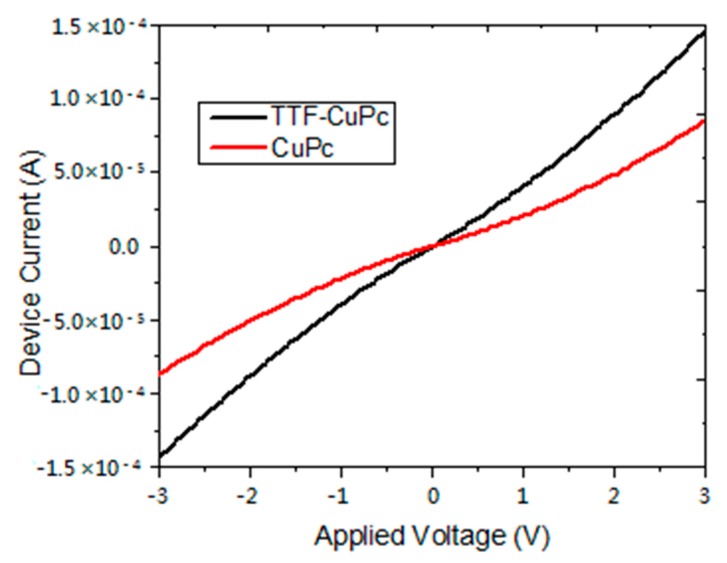
Current-voltage characteristics of *CuPc* device.

The role of the TTF-derivative dopant in the electrical properties of the MPc device is shown in Figure 8. A larger conductivity value can be seen in the TTF-containing CuPc device than in the TTF-less device. The TTF-ZnPc device shows a somewhat similar behavior as that of the TTF-CuPc. The increase in conductivity of the TTF-MPc compound may be explained by the presence of two different paths for the propagation of charge carriers along the TTF-derivative and MPcs compounds, in a way resembling that which occurs in similar charge-transfer compounds.

The size of the optical band gap, the electrical conductivities’ relative magnitudes and the feasibility of thin-film device preparation suggest the potential uses these materials may have in the fabrication of molecular electronic devices and heterostructures.

## 3. Experimental Section

MPcs and TTF derivative (4,4′,5,5′,6,6′,7,7′-octahydro dibenzo tetrathiafulvalene) were used in this study to obtain TTF-MPc (M = Cu, Zn) thin films; they were both purchased from Sigma-Aldrich (Saint Louis, MO, USA). All depositions were made on quartz substrates (Q-substrate), high resistivity (200 Ω·cm) single-crystalline, *n*-type silicon wafers (100) (Si-substrate) and ITO-coated glass slides. The Q-substrate was ultrasonically degreased for 5 min in 1,2-dichloroethane and then 5 min in methanol, and finally dried with a high-pressure flux of argon gas. The Si-substrate was chemically etched for 5 min with a *p* solution (10 mL HF, 15 mL HNO_3_ and 300 mL H_2_O) in order to remove the native oxide from the c-Si surface. The ITO-coated glasses were sonically cleaned in *n*-propanol, acetone, ethanol, deionized water and isopropyl alcohol (5 min in each solvent) and then dried in argon. The substrate temperatures were kept at 298 K during deposition. The evaporation equipment had two ports, each with its own crucible containing, respectively, TTF and MPc in a 1:1 stoichiometric ratio. All samples were obtained by the same vacuum deposition system, with two tungsten boats and rectangular substrates in a parallel arrangement within the evaporation equipment. The pressure in the vacuum chamber before the film deposition (1 × 10^−5^ Torr) and the evaporation rate were the same in all cases (2.2 Å/s). The thicknesses were monitored throughout the deposition processes using a quartz crystal monitor and were subsequently checked by means of profilometry. IR spectroscopy was used to determine the presence of the most representative functional groups and whether there were significant chemical changes in the materials during the thermal heat treatment. FT-IR measurements were obtained with a Nicolet iS5-FT spectrophotometer (Thermo Fisher Scientific Inc., Waltham, MA, USA) using KBr pellets for the powders and silicon wafers as substrates for the thin films. The optical absorption spectra of the composite films were measured in the wavelength range of 200–1000 nm on a *Unicam* spectrophotometer (Thermo Fisher Scientific Inc.), model *UV300*, with a quartz substrate. X-ray diffraction analysis of the films deposited on Si substrates was performed using the θ–2θ technique with a Rigaku Dmax 2100 diffractometer (Rigaku Corporation, Akishima-shi, Tokyo, Japan, Cu Kalpha1/Kalpha2 = 1.5406/1.5444 Å). Scan 2theta: 1°–80°, step size = 0.02° and step time = 0.3 s Film thickness and roughness values were determined by profilometry in a quartz substrate with a Bruker profilometer (Bruker Corporation, Billerica, MA, USA), DEKTAK XT model, with STYLUS, LIS 3, 2 μm RADIUS-Type B. The scanning electron micrographs and the chemical composition data were obtained on a Jeol JXA-8530F Field Emission Electron Probe Microanalyzer (JEOL Ltd., Akishima, Tokoyo, Japan). All devices used in these experiments had aluminum electrodes deposited by thermal evaporation on tungsten boats through an appropriate mask. The resulting area of each device was about 14.82 mm^2^. Figure 5 shows a schematic diagram of the device with a *glass/ITO/TTF_deriv_-MPc (M = Cu, Zn)/Al* structure. Samples were transferred to a vacuum system, and maintained under high vacuum for several hours prior to electrical measurements [4]. Electrical characterization of the devices was performed with a programmable voltage source, an auto-ranging Keithley 4200-SCS-PK1 pico-ammeter (Tektronix Inc., Beaverton, OR, USA) and a sensing station with lighting controller circuit Next Robotix (Comercializadora K Mox, S.A. de C.V., Benito Juárez, Distrito Federal, Mexico). The *I*-*V* characteristics were measured in the range −6 V to +6 V under illumination. All the samples were annealed at temperatures of 353 and 393 K for one hour in ordinary air conditions. The experiment involved two steps. First, the current due to hole-injection from positively-biased ITO was measured; and, second, the current due to hole-injection from Al was measured by reversing the polarity of the bias voltage [18].

## 4. Conclusions

The structural and optical properties of TTF_deriv_-MPc (M = Cu, Zn) thin films prepared by thermal vapor deposition and annealed at different temperatures have been investigated. The thermal evaporation process had no effect on the intra-molecular bonds, suggesting that the chemical composition of the films did not change. Thin-film morphology seems to depend on the molecular structure. The optical transitions were found to be of a non-direct nature. The optical band gap was calculated and the values for TTF_deriv_-CuPc and TTF_deriv_-ZnPc were found to be 1.4 and 1.7 eV, respectively. The different gap is related to factors such as the electronic configuration and differences in size of the metal atom, the overlap and the stacking within the TTF derivative molecules and the crystalline structure of the MPc.

TTF_deriv_-MPc (M = Cu, Zn) sandwich structures involving conductor-semiconductor interfaces have been fabricated using ITO and aluminum contact materials. A metal-semiconductor junction of the ohmic (non-rectifying) type was found, as expected for these *p*-type semiconductors and the work functions of the conducting electrodes. The results also show that an annealed-induced reduction in structural defects of the films led to significant improvements in the conductivity values. Doping with the TTF derivative led to enhanced conductivity values, which can be related to enhanced orbital overlap leading to conducting states within the band gaps. The dark current *J*-*V* characteristics of devices showed evidence of SCLC with exponential-trap-distribution mechanisms related to the existence of an ohmic contact with a limited potential barrier. From this work, TTF_deriv_-MPc (M = Cu, Zn) thin films show potentially useful electronic properties that may be applied to the production of ohmic electrode junctions and interconnects for molecular electronics applications.

## References

[1] Logothetidis S. (2008). Flexible organic electronic devices: Materials, process and applications. Mater. Sci. Eng. B.

[2] Rand B.P., Cheyns D., Vasseur K., Giebink N.C., Mothy S., Yi Y., Coropceanu V., Beljonne D., Cornil J., Brédas J.L. (2012). The impact of molecular orientation on the photovoltaic properties of a phthalocyanine/fullerene heterojunction. Adv. Funct. Mater..

[3] Rajesh K.R., Menon C.S. (2005). D.C. electrical and optical properties of vacuum-deposited organic semiconductor FePcCl thin films. Can. J. Phys..

[4] Shafai T.S., Anthopoulos T.D. (2001). Junction properties of nickel phthalocyanine thin film devices utilizing indium injecting electrodes. Thin Solid Films.

[5] Gravano S., Hassan A.K., Gould R.D. (1991). Effects of annealing on the trap distribution of cobalt phthalocyanine thin films. Int. J. Electron..

[6] Hassan A.K., Gould R.D. (1993). The interpretation of current density-voltage and activation energy measurements on freshly prepared and heat treated nickel phthalocyanine thin films. In. J. Electron..

[7] Peltekis N., Holland B.N., Krishnamurthy S., McGovern I.T., Poolton N.R.J., Patel S., McGuinness C. (2008). Electronic and optical properties of magnesium phthalocyanine (MgPc) solid films studied by soft X-ray excited optical luminescence and X-ray absorption spectroscopies. J. Am. Chem. Soc..

[8] Wang J.B., Li W.L., Chu B., Lee C.S., Su Z.S., Zhang G., Wu S.H., Yan F. (2011). High speed responsive near infrared photodetector focusing on 808 nm radiation using hexadecafluoro-copper-phthalocyanine as the acceptor. Org. Electron..

[9] Bialek B., Kim I.G., Lee J.I. (2002). Ab initio study of the electronic structure of nickel phthalocyanine-monolayer and bulk. Synth. Met..

[10] Bendikov M., Wudl F., Perepichka D.F. (2004). Tetrathiafulvalenes, oligoacenenes, and their buckministerfullerene derivatives: The brick and mortar of organic electronics. Chem. Rev..

[11] Farren C., Christensen C.A., FitzGerald S., Bryce M.R., Beeby A. (2002). Synthesis of novel Phthalocyanine-Tetrathiafulvalene hybrids; intramolecular fluorescence quenching related to molecular geometry. J. Org. Chem..

[12] Molas S., Caro J., Santiso J., Figueras A., Fraxedas J., Méziére C., Fourmigué M., Batail P. (2000). Thin molecular films of neutral tetrathiafulvalene-derivatives. J. Cryst. Growth.

[13] Wang C., Bryce M.R., Batsanov A.S., Stanley C.F., Beeby A., Howard J.A.K. (1997). Synthesis, spectroscopy and electrochemistry of phthalocyanine derivatives functionalized with four and eight peripheral tetrathiafulvalene units. J. Chem. Soc. Pekin Trans..

[14] Cook M.J., Cooke G., Jafari-Fini A. (1996). A liquid crystalline tetrathiafulvalenylphthalocyanine. Chem. Commun..

[15] Blower M.A., Bryce M.R., Devonport W. (1996). Synthesis and aggregation of a phthalocyanine symmetrically-functionalized with eight tetrathiafulvalene units. Adv. Mater..

[16] Wang C., Bryce M.R., Batsanov A.S., Howard J.A.K. (1997). Synthesis of pyrazinoporphyrazine derivatives functionalised with tetrathiafulvalene (ttf) units: X-ray crystal structures of two related ttf cyclophanes and two bis(1,3-dithiole-2-thione) intermediates. Chem. Eur. J..

[17] Nitschke C., O’Flaherty S.M., Kroell M., Strevens A., Maier S., Ruether M.G., Blau W.J., Grote J.G., Kaino T. Preparation and nonlinear optical properties of phthalocyanine nanocrystals. *Organic Photonic Materials and Devices V*, Proceedings of the SPIE.

[18] Mahapatro A.K., Ghosh S. (2007). Charge carrier transport in metal phthalocyanine based disordered thin films. J. Appl. Phys..

[19] Seoudi R., El-Bahy G.S., El Sayed Z.J. (2005). FTIR, TGA and DC electrical conductivity studies of phthalocyanine and its complexes. J. Mol. Struct..

[20] Sánchez M.E., Rivera M. (2014). Investigation of optical properties of annealed aluminum phthalocyanine derivatives thin films. J. Phys. Chem. Solids.

[21] El-Nahass M.M., Solimana H.S., Khalifab B.A., Soliman I.M. (2015). Structural and optical properties of nanocrystalline aluminum phthalocyanine chloride thin films. Mater. Sci. Semicond. Process..

[22] Melby L.R., Hartzler H.D., Sheppard W.A. (1974). An improved synthesis of tetrathiafulvalene. J. Org. Chem..

[23] El-Nahass M.M., Abd-El-Rahman K.F., Al-Ghamdi A.A., Asiri A.M. (2004). Optical properties of thermally evaporated tin-phthalocyanine dichloride thin films, SnPcCl2. Phys. B Condens. Matter.

[24] Robinet S., Clarisse C., Gauneau M., Salvi M. (1989). Spectroscopic and structural studies of scandium diphthalocyanine films. Thin Solid Films.

[25] El-Nahass M.M., Abd-El-Rahman K.F., Darwish A.A.A. (2005). Dispersion studies and electronic transitions in nickel phthalocyanine thin films. Opt. Laser Technol..

[26] Noguchi T., Gotoh K., Yamaguchi Y. (1991). Novel method to disperse ultrafine metal particles into polymer. J. Mater. Sci. Lett..

[27] Neghabi M., Zadsar M., Bagher Ghorashi S.M. (2014). Investigation of structural and optoelectronic properties of annealed nickel phthalocyanine thin films. Mater. Sci. Semicond. Process..

[28] Laidani N., Bartali R., Gottardi G., Anderle M., Cheyssac P. (2008). Optical absorption parameters of amorphous carbon films from Forouhi-Bloomer and Tauc-Lorentz models: A comparative study. J. Phys. Condens. Matter.

[29] El-Nahass M.M., Salam M.M., Ali H.A. (2005). Optical properties of thermally evaporated metal-free phthalocyanine (H_2_Pc) thin films. Int. J. Mod. Phys. B.

[30] El-Nahass M.M., Abd-El-Rahman K.F., Darwish A.A.A. (2005). Fourier-transform infrared and UV-vis spectroscopes of nickel phthalocyanine thin films. Mater. Chem. Phys..

[31] Seoudi R., El-Bahy G.S., El Sayed Z.A. (2006). Ultraviolet and visible spectroscopic studies of phthalocyanine and its complexes thin films. Opt. Mater..

[32] O’Leary S.K., Lim P.K. (1997). On determining the optical gap associated with an amorphous semiconductor: A generalization of the Tauc model. Solid State Commun..

[33] Mok T.M., O’Leary S.K. (2007). The dependence of the Tauc and Cody optical gaps associated with hydrogenated amorphous silicon on the film thickness: αl experimental limitations and the impact of curvature in the Tauc and Cody plots. J. Appl. Phys..

[34] Leontie L., Roman M., Brinza F., Podaru C., Rusu G.I. (2002). Electrical and optical properties of some new synthesized ylides in thin films. Synth. Met..

[35] Rodríguez-Gómez A., Sánchez-Hernández C.M., Fleitman-Levin I., Arenas-Alatorre J., Alonso-Huitrón J.C., Sánchez Vergara M.E. (2014). Optical absorption and visible photoluminescence from thin films of silicon phthalocyanine derivatives. Materials.

[36] Adachi S. (1999). Optical Properties of Crystalline and Amorphous Semiconductors: Materials and Fundamental Principle.

[37] Anthopoulos T.D., Shafai T.S. (2000). SCLC measurements in nickel phthalocyanine thin films. Phys. Status Solidi (a).

